# New insights on the potential effect of progesterone in Covid‐19: Anti‐inflammatory and immunosuppressive effects

**DOI:** 10.1002/iid3.1100

**Published:** 2023-11-28

**Authors:** Hayder M. Al‐Kuraishy, Thabat J. Al‐Maiahy, Ali I. Al‐Gareeb, Athanasios Alexiou, Marios Papadakis, Omnya Elhussieny, Hebatallah M. Saad, Gaber El‐Saber Batiha

**Affiliations:** ^1^ Department of Clinical Pharmacology and Therapeutic Medicine, College of Medicine Mustansiriyah University Baghdad Iraq; ^2^ Department of Gynecology and Obstetrics, College of Medicine Al‐Mustansiriyah University Baghdad Iraq; ^3^ University Centre for Research & Development Chandigarh University Mohali Punjab India; ^4^ Department of Science and Engineering Novel Global Community Educational Foundation Hebersham New South Wales Australia; ^5^ Department of Research & Development AFNP Med Wien Austria; ^6^ Department of Surgery II, University Hospital Witten‐Herdecke, Heusnerstrasse 40 University of Witten‐Herdecke Wuppertal Germany; ^7^ Department of Histology and Cytology, Faculty of Veterinary Medicine Matrouh University Marsa Matruh Egypt; ^8^ Department of Pathology, Faculty of Veterinary Medicine Matrouh University Marsa Matruh Egypt; ^9^ Department of Pharmacology and Therapeutics, Faculty of Veterinary Medicine Damanhour University, Damanhour AlBeheira Egypt

**Keywords:** COVID‐19, cytokines, immunological tolerance, progesterone

## Abstract

**Background**: Coronavirus disease 2019 (COVID‐19) is a pandemic disease caused by severe acute respiratory syndrome CoV type 2 (SARS‐CoV‐2). COVID‐19 is higher in men than women and sex hormones have immune‐modulator effects during different viral infections, including SARS‐CoV‐2 infection. One of the essential sex hormones is progesterone (P4). **Aims**: This review aimed to reveal the association between P4 and Covid‐19. **Results and Discussion**: The possible role of P4 in COVID‐19 could be beneficial through the modulation of inflammatory signaling pathways, induction of the release of anti‐inflammatory cytokines, and inhibition release of pro‐inflammatory cytokines. P4 stimulates skew of naïve T cells from inflammatory Th1 toward anti‐inflammatory Th2 with activation release of anti‐inflammatory cytokines, and activation of regulatory T cells (Treg) with decreased interferon‐gamma production that increased during SARS‐CoV‐2 infection. In addition, P4 is regarded as a potent antagonist of mineralocorticoid receptor (MR), it could reduce MRs that were activated by stimulated aldosterone from high AngII during SARS‐CoV‐2. P4 active metabolite allopregnanolone is regarded as a neurosteroid that acts as a positive modulator of γ‐aminobutyric acid (GABA_A_) so it may reduce neuropsychiatric manifestations and dysautonomia in COVID‐19 patients. **Conclusion**: Taken together, the anti‐inflammatory and immunomodulatory properties of P4 may improve central and peripheral complications in COVID‐19.

## Graphical abstract

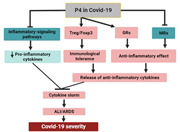

## 1 INTRODUCTION

Severe acute respiratory syndrome CoV type 2 (SARS‐CoV‐2) was initially recognized as a probable cause of coronavirus disease 2019 (COVID‐19) in China in late December 2019. SARS‐CoV‐2 exploits angiotensin‐converting enzyme type 2 (ACE2) and other receptors to bind host cells. SARS‐CoV‐2, when it binds ACE2, persuades a sequence of inflammatory events causing cell injury, hyper‐inflammation, and cytopathic effects.^1^ ACE2 is largely distributed in various cellular systems, mainly in pulmonary alveolar cells, neurons, testes, and enterocytes.^2^ The clinical presentation of Covid‐19 is usually asymptomatic in most cases. However, some of the infected cases developed acute lung injury (ALI) and acute respiratory distress syndrome (ARDS) that need intensive care admission. ACE2 is a peptidase metabolizes vasoconstrictor angiotensin II (AngII) to the vasodilator Ang1‐7. Hence, downregulation of ACE2 during SARS‐CoV‐2 infection induces vasoconstriction and progression of endothelial dysfunction (ED), oxidative stress, and inflammatory disorders causing hypoxia hypo‐perfusion and cytokine storm leading to cardio‐pulmonary complications.^3^

It has been reported that hospitalizations and mortality from COVID‐19 are higher in men than women due to less susceptibility of women to viral infections with low immune response and renin‐angiotensin system (RAS) activity.^4^ The claim about COVID‐19 disease incidence and disease severity in men and women is based on a mouse model. The high mortality observed in COVID‐19 patients may be related to unrecognized pulmonary embolism, pulmonary thrombosis, or other underlying cardiovascular diseases. Recent data have highlighted that the mortality rate of COVID‐19 seems to be higher in male patients compared to women.^5^ A study carried out by Liu et al.^6^ on 4880 patients with respiratory symptoms or close contact with COVID‐19 patients in a hospital in Wuhan showed that there was a significantly higher rate of positivity to SARS‐CoV‐2 in males and the elderly population (>70 years), although only age was recognized as a risk factor. Death rates in all age groups are twice as high for men as for women. Small changes in contact rates at working and young ages have a considerable effect on infections and mortality in old age, with elderly men always at higher risk of infection and mortality.^7^

There is mounting evidence that sex hormones affect RAS and the expression of ACE2 since ovariectomy increases the expression of ACE2 in females.^8^ Sex hormones have immune‐modulator effects during different viral infections, including SARS‐CoV‐2 infection.^8^ Also, sex hormones are pretentious by COVID‐19 due to dysregulation of the hypothalamic‐pituitary‐gonadal axis with subsequent reduction of circulating sex hormones.^9^ One of the essential sex hormones is progesterone (P4), which has immunomodulatory effects in various viral infections and autoimmune diseases.^10^ Therefore, this review tries to reveal the association between P4 and Covid‐19.

### 1.1 Progesterone overview

**Figure 1:**
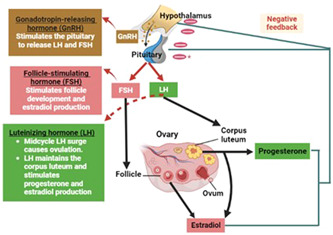
Secretion of progesterone (P4): Gonadotrophin‐releasing hormone (GnRH) from the hypothalamus stimulates the release of follicle‐stimulating hormone (FSH) and luteinizing hormone (LH) from the anterior pituitary. FSH induces folliculogenesis and release of estrogen while LH induces ovulation and formation of the corpus luteum, which secretes P4. Both P4 and estrogen cause negative feedback inhibition of GnRH, FSH, and LH from the hypothalamus and anterior pituitary, respectively.

Progesterone (P4) is a sex hormone of endogenous steroids involved in controlling the menstrual cycle, pregnancy, and embryogenesis.^11^ P4 acts as an intermediate in producing endogenous steroids like corticosteroids and sex hormones.^11^ P4 is secreted from the ovary and to a lesser extent from the adrenal cortex under the effect of gonadotropin hormones from the anterior pituitary. P4 secretion is peaked at the mid‐luteal phase and rapidly reduced in the late luteal phase^12^ (Figure 1).

P4, in combination with estrogen, is used as hormone replacement therapy in menopause and as contraceptives during the fertility period. George and Willard discovered P4 in 1929, and in 1932 pure P4 was isolated from the corpus luteum. Adolf Butenandt in 1934 described the chemical structure of P4, and it was first prescribed for clinical use in 1934.^13^

**Figure 2:**
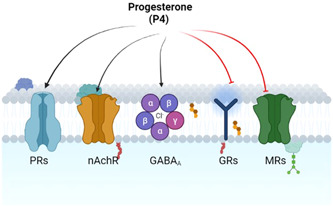
Progesterone (P4) and receptor effects: P4 activates progesterone receptors (PRs), nicotinic acetylcholine receptor (nAchR), and inhibits glucocorticoid receptors (GRs) and mineralocorticoid receptors (MRs). P4 is regarded as a positive allosteric of γ‐aminobutyric acid type A (GABAA) receptors.

P4 is regarded as a strong agonist of nuclear progesterone receptor (PR) and membrane progesterone receptors (mPRs). In addition, P4 acts as a ligand for progesterone receptor component 1 (PGRMC1), regulating body metabolism, tumor growth, and neuron viability.^14^ However, P4 is considered an antagonist for mineralocorticoid receptors (MRs)^15^ and sigma receptors with an allosteric modulator of nicotinic acetylcholine receptor (nAchR).^16^ In addition, P4 acts as an antagonist of glucocorticoid receptors (GRs),^17^ and P4 metabolites like allopregnanolone and 5α‐dihydro‐progesterone operate as neurosteroids and act as positive allosteric modulators of γ‐aminobutyric acid type A (GABA_A_) receptors.^18^ Likewise, P4 metabolite 5β‐dihydro‐progesterone activates the pregnane X receptor (PXR), which is regarded as xenobiotic and steroid‐sensing nuclear receptors involved in the detoxification of toxic agents from the body^19^ (Figure 2).

P4 has a moderate plasma protein binding activity, about 50%–54% for albumin and 43%–48% for transcortin. P4 is metabolized by hydroxysteroid dehydrogenase in hepatic and extra‐hepatic tissues like the skin and brain. Both 5β‐reductase and 5α‐reductase are the major pathways in the metabolism of P4 into inactive metabolites which are excreted by kidneys.^20^

A high P4 serum level reduces aldosterone activity resulting in a reduction of extracellular fluid by natriuresis, though a low P4 serum level causes fluid retention due to compensatory elevation of aldosterone.^21^ Moreover, P4 is involved in the regulation of different pathophysiological changes. It improves libido in females, increases attitude toward homosexuality in both women and men,^22^ neuroprotective effects through modulation of neurotransmitters and activation of nAChRs.^23^ Therefore, P4 is effective in traumatic brain injury by inhibiting voltage‐dependent Ca^+2^ channels, reducing excitotoxicity, upregulation of GABA, and attenuation of neuronal apoptosis. In addition, P4 attenuates neurodegeneration by antioxidant effects and enhancement of remyelination of injured neurons.^24^

Prolong use of P4 is associated with the development of many adverse effects which are classified as very common (10% or more) including breast enlargement (40%), breast tenderness (27%), perineal pain female (17%), breast pain (16%), nocturia (13%), urinary problems (11%), vaginal discharge (11%), Common (1% to 10%) including ovarian hyperstimulation syndrome, breast pain, vaginal dryness, pruritus genital, uterine spasm, vaginal bleeding, altered periods, amenorrhea, intercurrent bleeding, and uncommon (0.1%–1%) including vulvovaginal disorders, vaginal mycosis, breast disorders, pollakiuria, incontinence, ovarian enlargement, pelvic pain, vulvovaginal pruritus, galactorrhea.^20^, ^23^, ^24^ There is no acute toxicity, and the risk of deep vein thrombosis (DVT) or pulmonary embolism associated with progesterone treatment exists but is minimal. First, the risk of DVT is associated with chronic hormone therapy, over months or years, not following acute hormone therapy of several days.^20^, ^21^, ^23^, ^24^

### 1.2 Progesterone and immune response

PRs are widely distributed in the immune cells and play a crucial role in the immunomodulation effects.^25^ Of note, mPRs including mPRα, mPRβ, and mPRγ are expressed in peripheral blood leukocytes, T lymphocytes, and immortalized T cells (Jurkat cells). Expression of the mPRα messenger RNA was upregulated in the luteal phase of the menstrual cycle in a cluster of differentiation (CD)^8+^, but not in CD^4+^, T lymphocytes. P4 binding to T and Jurkat cell membranes of steroid membrane receptors. P4 activated an inhibitory G‐protein suggesting that mPRs are coupled to inhibitory G‐protein in Jurkat cells. These results suggest a potential novel mechanism for P4 immunoregulatory function through activation of mPRs.^26^, ^27^ P4‐induced blocking factor (PIBF) is a protein secreted by lymphocytes exposed to P4. P4 and PIBF have immunomodulatory effects on peripheral CD4^+^ T cells during normal pregnancy. As well, mPRs may correlate with the immunomodulatory properties of P4 on T cells. Variation in the expression of mPRs may influence P4 regulatory performance during pregnancy. PIBF increases in pregnant normal women compared to women who have experienced abortion.^26^, ^27^ P4 inhibits innate immune response by suppressing the activity of dendritic cells and macrophages by inhibiting the release of pro‐inflammatory cytokines by interfering with nuclear factor kappa B (NF‐κB).^28^ P4 through activation of GRs, attenuates the expression of IL‐1β and the down‐streaming of inflammatory cyclooxygenase‐2 (COX‐2) by inhibiting mitogen‐activated protein kinase (MAPK) signaling pathways.^29^ It has been shown that the anti‐inflammatory of P4 is mediated by GRs rather than by PRs. Moreover, P4 inhibits the expression of toll‐like receptors (TLRs) 3 and 4 and the release of IL‐6, tumor necrosis factor‐α (TNF‐α), and IL‐1β as well as the expression of CD86 and CD80.^30^ Likewise, P4 inhibits T cell and macrophage activation in animal model studies by inhibiting the production of nitric oxide.^30^ P4 stimulates skew of naïve T cells from inflammatory Th1 toward anti‐inflammatory Th2 with activation release of anti‐inflammatory cytokines (IL‐4, IL5, IL‐10, IL‐13, and IL‐27), activation of regulatory T cells (Treg) with decreased interferon‐gamma (INF‐γ) production.^31^ Similarly, P4 inhibits the activation of natural killer and eosinophilic cells.^31^ P4 improves Treg through modulation of signal transducer and activator transcription 3 (STAT3).^32^ P4 improves STAT5 activation in response to the IL‐2 effect, but it reduces STAT3 activation in response to IL‐6.^32^ This finding indicates the selective effect of P4 on the development of Treg compared to Th17. Indeed, P4 inhibits the function and proliferation of B cells through a reduced expression of CD86 and CD80 limiting antigen presentation and antibody production.^33^ P4 has a negative regulatory effect on bone marrow cell proliferation.^33^ Therefore, P4 improves the anti‐inflammatory, immune response by inhibiting pro‐inflammatory cytokines and activating anti‐inflammatory cytokines.

Moreover, P4 reduces the risk of autoimmune diseases by counteracting the effects of estrogen on the immune cells.^34^ Polikarpova et al.^35^ illustrated that P4 and its analogs have anti‐inflammatory and pro‐inflammatory effects at the same time depending on the phenotype of immune cells. Therefore, P4 may inhibit or stimulate the immune function depending on the underlying inflammatory state. Hughes^36^ found that P4 at a low physiological level enhances the INF‐α pathway, which is vital in the pathogenesis of systemic lupus erythematosus. During pregnancy, P4 attenuates the progression of autoimmune diseases like multiple sclerosis and rheumatoid arthritis through inhibition Th1/Th17 axis.^36^ P4 reduces inflammatory cytokines (IL‐2, IL‐17) and increases anti‐inflammatory cytokines IL‐10 in experimental autoimmune encephalomyelitis.^37^ Additionally, P4 improves immune tolerance by increasing forkhead box P3 (FOXP3) Treg cells in cord blood cells^38^; therefore, P4 can attenuate the progression of autoimmunity by inhibiting T cell activation.

Furthermore, P4 active metabolites like allopregnanolone are mainly produced in the brain and act as a positive modulator of GABA_A_.^39^ Pregnenolone, which is devoid of action on GABA_A_, can inhibit toll‐like receptor 4 (TLR4) signaling.^40^ These active metabolites inhibit TLR4 signaling, which involves activating macrophage adaptor protein 2 and myeloid differentiation 88 (MyD88) pathways in the brain.^41^ Allopregnanolone and pregnenolone also increase the degradation of TLR4 and attenuate the release of pro‐inflammatory signaling pathways during immune activation.^41^ Thus, P4 active metabolites inhibit the MyD88‐TLR4 signaling pathway and attenuate inflammatory reactions. Indeed, P4 has an immunosuppressive effect by downregulating the INF pathway, inhibiting the STAT1/STAT2 pathway, augmenting Treg cells, and inhibiting antigen‐presenting cells.^42^, ^43^ This observation indicates that P4 may increase the escape of tumor cells from the immune system with subsequent tumor progression. Various human and animal model studies illustrated that P4 promotes immune homeostasis by enhancing anti‐inflammatory immune response, though, under the effect of P4, the dendritic cells and macrophages may produce a small amount of pro‐inflammatory cytokines, including IL‐6 and IL‐1β.^44^

**Figure 3:**
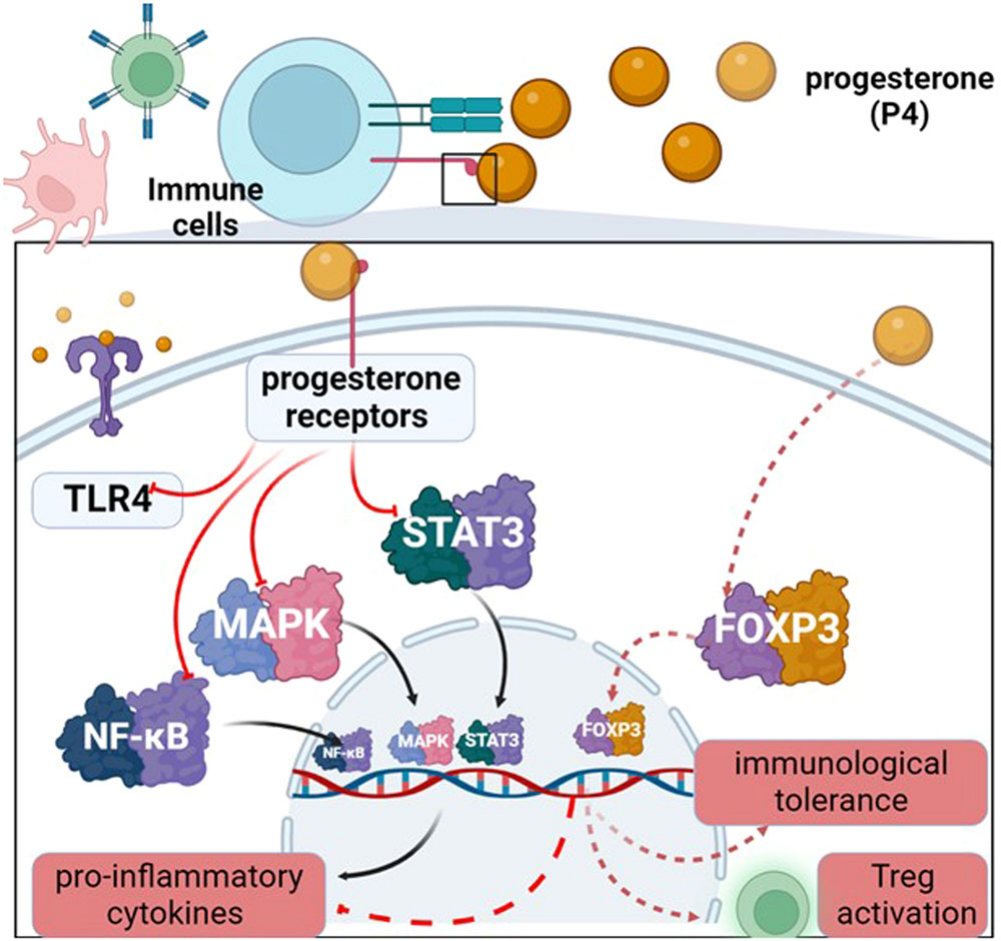
Immunological effects of progesterone (P4): P4 through progesterone receptors on the immune cells inhibits mitogen‐activated protein kinase (MAPK), nuclear factor kappa B (NF‐κB) signal transducer and activator transcription 3 (STAT3), and expression of toll‐like receptor 4 (TLR4), while P4 increases forkhead box P3 (FOXP3) leading to inhibition release of pro‐inflammatory cytokines and improves immunological tolerance.

These verdicts revealed the immunological effects of P4, which is primarily anti‐inflammatory, preventing immuno‐inflammatory complications in different inflammatory and autoimmune disorders (Figure 3).

### 1.3 Progesterone and viral infections

#### 1.3.1 Influenza viral infection

It has been reported that P4 had differential effects on the outcomes of viral infections that could be beneficial or harmful. In influenza, A virus infection that infects epithelial cells of the respiratory system can induce immune cell activation and release of pro‐inflammatory cytokines.^45^ These inflammatory changes trigger tissue damage in the respiratory tract, mainly in the lung with the development of ALI.^45^ In this sense, many immune and epithelial cells produce epidermal growth factor (EGF), which is intricate in tissue repair following ALI and tissue injury in different viral infections.^46^ Hall et al.^47^ revealed that P4 could attenuate immune response and improve tissue repair and outcomes in influenza A virus infection. However, experimental studies found that young females had severe and worse outcomes following influenza A virus infection than young adult males due to suppression of the release of sex hormones, mainly P4.^48^ P4 improves pulmonary function and promotes lung repair following ALI and inflammation, with induction of rapid recovery in a female with influenza A virus infection.^48^ This effect is mainly mediated by promoting the release of EGF, tumor growth factor‐beta (TGF‐β), induction of the development of Treg, and regulation of the Th17 CD39+ population in pulmonary tissues that maintain the protective effects during this viral infection.^49^ Also, P4 increases neutralizing antibody titer against influenza A virus with protection from severe outcomes but increases susceptibility to severe outcomes in heterogeneous infections with influenza A virus.^49^ Taken together, P4 has differential immunomodulatory effects in influenza A virus infections by improving the expression of Treg Th17 which attenuates lung inflammation in in influenza A virus infections.^50^

Similarly, P4 induces the expression of IL‐22, which promotes the regeneration of pulmonary epithelial cells following infection with the influenza A virus.^50^ Enomoto et al.^51^ illustrated that P4 improves lung repair and regeneration through induction of the expression and the release of EGF and amphiregulin in the pulmonary airway of hospitalized asthmatic children. Therefore, the administration of recombinant amphiregulin protects from the development of severe outcomes of influenza A virus infection by reducing hypothermia‐induced airway protein leakage.^52^ Amphiregulin is mainly produced from epithelial and immune cells, including Treg cells expressing PRs that have protective effects against influenza A virus infection and are intricate in lung repair.^53^ These observations suggest the protective effect of P4 against influenza A virus infection and could be a potential therapeutic modality in treating such infections in menopause.

## 2 HUMAN IMMUNE DEFICIENCY VIRUS 1 (HIV‐1)

Different studies documented that the acquisition of HIV‐1 infection in females was increased in females during the luteal phase of the menstrual cycle when P4 is highest and during the use of hormonal contraceptives.^54^ However, a pilot study conducted by Boodhram et al.^55^ on 39 HIV‐positive women on oral contraceptive pills compared to 36 women on oral contraceptive pills uninfected with HIV, showed that P4 serum level did not differ significantly, suggesting that inhibition of P4 by oral contraceptive was not associated with acquisition risk of HIV‐1 infection. P4 increases the expression of chemokine receptors with the alteration of cytokine response of immune cells in HIV‐1 infection by reducing the production of INF‐γ, TNF‐α, IL‐2, IL‐6, and IL‐17.^56^ Likewise, a higher level of P4 promotes the synthesis of type I INF from plasmacytoid dendritic cells via the activation of TLR7 by HIV‐1.^57^

On the other hand, there is a close relationship between pregnancy and the risk of HIV‐1 infection regarding the potential role of P4.^58^ HIV‐1 co‐receptors CXCR4 and CCR5 on the immune cells are higher during pregnancy due to the positive effect of P4 on the expression of these receptors.^59^ This finding suggests that a high P4 serum level during pregnancy increases the risk for HIV‐1 infection. Asin et al.^60^ demonstrated that the integration and replication of HIV‐1 are reduced in the mid‐secretory phase, and increased in the mid‐proliferative phase of the menstrual cycle by direct alteration of the activity of TLR by P4 and estrogen. Conversely, Munoz et al.^61^ found that P4 blocks HIV‐1 replication in trophoblastic tissues by inhibiting TNF‐α, which is necessary for optimal viral replication.

Herein, there are potential controversies regarding the role of P4 in the acquisition risk of HIV‐1 replication during the reproductive period and hormonal replacement therapy during menopause.

### 2.1 Hepatitis viral infections

Infection with hepatitis E virus (HEV) is linked with high feto‐maternal mortality; a prospective study included 100 pregnant women with acute HEV and 43 with fulminant HEV compared to 50 healthy pregnant women regarding the expression of PRs and PIBF showed that viral load was higher in patients with fulminant HEV compared to acute HEV.^62^ PRs and PIBF were lower in HEV compared to healthy controls. The IL‐12/1L‐10 ratio was also higher in fulminant HEV and feto‐maternal mortality.^62^ These findings suggest that PRs and PIBF are reduced in HEV and linked with feto‐maternal complications. Debes et al.^63^ proposed that mutation in PRs and seropositivity for HEV in patients with liver transplant recipients is associated with distorted cytokine levels. Indeed, P4 mediates the replication of HEV in human liver cells.^64^

Concerning the role of P4 in HBV, it induces the production of pro‐inflammatory cytokines in patients with chronic HBV infection.^65^ Therefore, P4 may aggravate HBV‐induced liver injury by triggering the accumulation of monocytes/macrophages with the induction release of pro‐inflammatory cytokines. Furthermore, the downregulation of the P4 PRMC‐1 receptor is associated with the progression of hepatocellular carcinoma in patients with HBV.^66^ Buettner et al.^67^ revealed that the incidence of HBV and risk of hepatocellular carcinoma were lower in females than males due to the protective immune‐modulatory effects of P4.

Regarding HCV, P4 inhibits INF signaling by suppressing the expression of TLR7 in peripheral blood mononuclear cells in patients with HCV.^68^ Higher P4 concentration in the second and third trimesters of pregnancy promotes HCV RNA levels and the development of chronic HCV.^69^ Sarkar et al.^70^ found that sex hormones are distorted in women with chronic HCV complicating with cirrhosis. HCV complicating with cirrhosis is evident in the development of amenorrhea and feminization in females and males, respectively.^70^ Both follicular stimulating hormone and sex hormone‐binding globulin were reduced in patients with chronic HCV leading to high estrogen and low P4 serum levels.^70^

These explanations revealed a controversial role of P4 in different hepatitis viral infections, which could be beneficial or harmful.

### 2.2 Other viral infections

Moreover, P4 increases the susceptibility to herpes simplex virus infection (HSV) and reduces protection after HSV vaccination.^71^ P4 reactivates HSV infection with reduced activation of specific virus‐CD^8+^, cytokine production, and impairment of expression of co‐stimulatory molecules on the dendritic cells.^71^ Similarly, P4 promotes HSV2 infection in the genital mucosa by activating mucosal permeability and recruitment of inflammatory cells through the activation release of IL‐1β. Likewise, P4 inhibits the response and production of anti‐HSV2 during HSV2 vaccination.^72^ In addition, norethisterone enanthate and depot medroxyprogesterone acetate (DMPA) increase the risk of HSV and HIV‐1 infections by weakening genital mucosal barriers by reducing mucosal desmoglein‐1.^73^ These findings pointed out that P4 and its analogs increase the risk of different HSV infections.

On the other hand, viral infections may affect the P4 level by different mechanisms. For example, the proliferation of Zika virus (ZV) in the antral follicles promotes follicle degeneration with subsequent distortion of ovarian steroidogenesis and the development of ovarian failure.^74^ These pathological changes lead to the inhibition release of P4 and other ovarian hormones. In addition, protease inhibitors like ritonavir, atazanavir, and lopinavir inhibit trophoblastic P4 production resulting in low circulating P4 during pregnancy and subsequent feto‐maternal complications.^75^

### 2.3 Progesterone and Covid‐19

It has been shown that P4 improves innate immune response and mitigates the release of pro‐inflammatory cytokines by promoting immune tolerance and T cells‐mediated anti‐inflammatory response.^76^, ^77^, ^78^ Therefore, treatment with P4 may mitigate the development of cytokine storms in Covid‐19 patients.^79^ In addition, a critical advantage of P4 therapy in COVID‐19 is related to the blunting of innate immune response with the activation of B cells for the production of antibodies.^79^ At this instant, P4 may prevent an exaggerated immune response against SARS‐CoV‐2 in the initial immune response phase to attenuate the development of cytokine storm. The hyperimmune response in the initial phase is suppressed by P4 and stimulated by estrogen, though immunosuppressant effects of dexamethasone improve 28‐day mortality in COVID‐19 patients.^80^ These observations emphasize the valuable role of immunosuppressive agents like P4 in the management of COVID‐19. Therefore, low female susceptibility to SARS‐CoV‐2 infection and COVID‐19 complications is mainly related to the potential anti‐inflammatory and immunosuppressive effects of P4.^81^ Thus, P4 counteracts the immune‐stimulatory effects of estrogen.^82^ Furthermore, P4 promotes the development of Treg T cells with a reduction of Th17 and production of INF‐γ that controls viral replication and associated immune‐inflammatory changes.^83^ Notably, Treg T cells are severely reduced in SARS‐CoV‐2 infection and linked with Covid‐19 severity. Liu et al.^84^ proposed that induction of immunosuppressive Treg T cells can attenuate SARS‐CoV‐2 infection‐induced lung inflammation and ALI. Besides, Foxp3 is regarded as a natural regulator of Treg and adaptive immune response and/or immune tolerance.^85^ Foxp3 is reduced in patients with severe COVID‐19 due to the downregulation of Treg cells.^85^ P4 regulates immune tolerance by rising forkhead box P3 (FOXP3) Treg cells in immune cells.^38^ Therefore, P4 could reduce immune dysregulation by improving FOXP3/Treg in Covid‐19.

In addition, Th17 cells, which produce IL‐17, IL‐21, and IL‐22, can induce neutrophil recruitment, systemic inflammation, upregulation of fibrinogen, and development of cytokine storm. High pro‐inflammatory cytokines and cytokine storms in COVID‐19 patients promote the skewing of naïve T cells toward Th1 and Th17 with successive inflammatory changes. Hu et al.^86^ found that low circulating INF‐γ is regarded as a risk factor for the development of lung fibrosis in COVID‐19 patients. P4 modulates immune response by regulating the production of INF‐γ.^83^ In this sense, P4 therapy could be beneficial against SARS‐CoV‐2 infection and associated hyperinflammation/cytokine storm through mitigation of Treg cells, Th17, and INF‐γ immune response. The roles of immune cells in pathological processes and the possible avenues for induction of immunosuppressive T regulatory cells attenuating lung inflammation due to viral infection have been illustrated.^84^ Treg cells act against viral pneumonia indirectly by suppressing the immune response. Treg cells likely use multiple mechanisms of suppression including secreted proteins and cell surface molecules. Three secreted proteins are highly identified with suppression of Tregs, including TGF‐β, IL‐10, and IL‐35.^87^ Furthermore, in inhibition of inflammatory responses following virus infection, Tregs could promote tissue repair by expression of amphiregulin, which does not depend on the immunosuppressive activity of Treg.^88^ These biological characteristics suggested that Treg cells may have multiple antivirus protection mechanisms against SARS‐CoV‐2 infection.

Preclinical findings indicated that P4 rescued the body weight loss of the SARS‐CoV‐2‐infected hamsters in a dose‐dependent manner.^89^ A randomized, open‐labeled controlled clinical trial involved 42 COVID‐19 patients, 22 on the standard therapy alone and 20 patients on standard therapy plus P4 100 mg/twice daily illustrated that P4 improved oxygenation and hospitalization.^90^ Another clinical trial illustrated the therapeutic effect of P4 treatment in the management of severely hospitalized COVID‐19 patients.^91^ In addition, norelgestromin and ethinylestradiol transdermal patches could be safe and effective treatments for moderate and severe COVID‐19 patients.^92^ A systematic review showed that hormone replacement therapy was positively associated with reducing COVID‐19 symptoms.^93^ There is some evidence that P4 contributes to gender differences related to COVID‐19, even though the majority of studies looked at the potential role of estrogen against COVID‐19. Ghandehari et al.^90^ in a randomized, controlled pilot trial, demonstrated that P4 at a dose of 100 mg added to standard of care could represent an effective and safe method for treatment in men hospitalized with confirmed moderate to severe COVID‐19. As well, higher endogenous levels of progesterone may protect women from progressing to severe illness in COVID‐19. Altogether, P4 is an important biological factor that may modulate the gender bias of SARS‐CoV‐2 infection and pathogenesis, and might be able to serve as a potential therapeutic agent for COVID‐19.^89^ More clinical investigations regarding hormone supplements using P4 are needed to validate the therapeutic protective role of this hormone against COVID‐19 infection.

These findings suggest that P4 therapy is effective in the management of severe hypoxemic Covid‐19 patients. The underlying protective effect of P4 in COVID‐19 is mainly due to the inhibition release of pro‐inflammatory cytokines with augmentation of anti‐inflammatory cytokines through inhibition of IL‐6 and increment of IL‐10, respectively.^90^

Of note, a low dose of P4 inhibits the NF‐κB signaling pathway and prevents the development of endometriosis.^94^ In addition, P4 hampers innate immune response by repressing the action of dendritic cells and macrophages with attenuation release of pro‐inflammatory cytokines via interfering with NF‐κB.^28^ Activation of the NF‐κB signaling pathway is associated with the development of hyperimmune stimulation, the release of pro‐inflammatory cytokines, and the development of ALI/ARDS in COVID‐19.^95^ Thus, P4, through inhibition of the NF‐κB signaling pathway, could be a potential candidate in treating SARS‐CoV‐2 infection‐induced ALI/ARDS. Furthermore, it has been reported that prenatal P4 deprivation impairs lung alveolar formation and fluid clearance during the postnatal period, suggesting the protective effect of P4 against ALI.

Indeed, P4, through stimulation of GRs, reduces the expression of IL‐1β and COX‐2 by inhibiting MAPK signaling pathways.^29^ Hierwerger et al.^96^ observed that P4 promotes T cell apoptosis by activating T cell GRs by P4. In COVID‐19, due to underlying pro‐inflammatory reactions, there is increased expression of GRs in critically ill patients due to steroid resistance.^97^ Most of the P4 immune effects are succeeded through the PR, which is widely distributed on the immune cells and acts by the trans‐repression manner to inhibit pro‐inflammatory gene transcription.^98^ However, owing to the considerable pleiotropism, P4 can bind and activate other steroid receptors, preferably GR and MR, but with less potency than the original ligands.^98^ Knowing the physiological effects of the corresponding hormones, some potentially protective effects of P4 during SARS‐CoV‐2 infection could be also accredited to the P4‐mediated activation of GRs and MRs. The beneficent effect of GRs activation is in line with the reduction of mortality in dexamethasone‐treated severe Covid‐19 patients.^80^ A recent study on animal models suggested that P4 immunomodulatory impact on T cell response is achieved strictly by means of GRs.^31^

Besides, SARS‐CoV‐2 infection is associated with the activation of MAPK due to the downregulation of ACE2 and upregulation of AngII. Also, SARS‐CoV‐2 can directly activate MAPK to promote its replication. Activated MAPK induces vasoconstriction and immuno‐thrombosis with subsequent COVID‐19 complications, including lung fibrosis and ALI/ARDS.^99^ Therefore, P4, through selective activation of GRs (partial agonist) and suppression of MAPK, could reduce hyper‐inflammation and cardio‐pulmonary complications with modulation of glucocorticoid sensitivity in Covid‐19. Even though the role of P4 in the context of SARS‐CoV‐2 infection has been somewhat neglected, the available findings strongly indicate P4's importance in establishing sex disparity of the Covid‐19 course and outcome. Furthermore, cumulative evidence points to the protective role of P4 during SARS‐CoV‐2 infection nominating it for drug repurposing consideration.

Notably, P4 is regarded as a potent antagonist of MR activated by stimulated aldosterone from high AngII during SARS‐CoV‐2 infection. Aldosterone exerts immunological effects through expressing MRs on the immune cells.^100^ Furthermore, activation of MRs induces macrophage polarization toward classical inflammatory M1 phenotype that triggers the release of pro‐inflammatory cytokines with noteworthy development of Th17 immune response.^101^ Therefore, MR antagonist spironolactone can attenuate hyper‐inflammation, macrophage polarization, and deregulated RAS in Covid‐19.^89^, ^93^, ^102^, ^103^ In the bargain, since P4 is a potent MRs antagonist, it could reduce MRs–induced immunological disorders and hyper‐inflammation in COVID‐19.

Furthermore, P4 hinders the expression of TLR4 and CD80/CD86.^30^ Of note, SARS‐CoV‐2 directly activates TLR4 with the activation of the NF‐κB signaling pathway and the release of pro‐inflammatory cytokine and the development of cytokine storm. Thus, TLR4 antagonists like eritoran are effective against different inflammatory disorders like rheumatoid arthritis in humans.^104^ Therefore, targeting TLR4 by P4 might be an effective therapeutic opportunity in managing SARS‐CoV‐2 infection‐induced immuno‐inflammatory complications as P4 and its metabolites inhibit the MyD88‐TLR4 signaling pathway.^41^

Likewise, nod‐like receptor pyrin 3 (NLRP3) inflammasome is highly activated in SARS‐CoV‐2 infection and, together with TLR4, MAPK, and NF‐κB signaling pathways, leading to macrophage activation and release of pro‐inflammatory cytokines.^105^ It has been found that P4 with vitamin D attenuates TLR4/MAPK/NF‐κB/NLRP3 inflammasome in monocytes. Herein, P4, through inhibition of inflammatory signaling pathways, may reduce the inflammatory changes in SARS‐CoV‐2 infection and could be a therapeutic option in managing patients with severe COVID‐19.

Interestingly, P4 reduces STAT3 activation in response to IL‐6.^38^ SARS‐CoV‐2 viral components induce activation of STAT3 due to the dysfunction of STAT1.^106^ In turn, STAT3 activates plasminogen activator inhibitor 1 (PAI‐1), causing coagulopathy and micropulmonary thrombosis. Hyper‐activated and overproduced PAI‐1 binds TLR4 on macrophages causing secretion of chemokines and pro‐inflammatory cytokines, leading to ED and development of ALI/ARDS and hypoxia with further activation release of PAI.^107^ Therefore, there is a positive feedback loop between STAT3 and PAI‐1 in SARS‐CoV‐2 infection‐induced ALI/ARDS pathogenesis. Inhibition of STAT3 by P4 may repress the progression of this inflammatory feedback loop in SARS‐CoV‐2 infection and prevent immune‐thrombosis consequences. Sandberg et al.^108^ found that P4 could stabilize PAI‐1 and prevent its activation. Therefore, P4, through its potential effects on STAT3 and PAI‐1 pathway, P4 could reduce the risk of hyperinflammation and thrombosis linked with severe SARS‐CoV‐2 infection.

Interestingly, autophagy is an essential intracellular mechanism for eliminating and recycling toxic unfolding proteins during inflammatory diseases, including COVID‐19.^5^ SARS‐CoV‐2 can hijack cell auto‐phagosomes and use them as replicative niches to down‐regulate cell anti‐viral mechanisms.^109^ Both activated autophagy and SARS‐CoV‐2 can stimulate the mechanistic target of the rapamycin (mTOR) pathway, which improves viral survival and induces hyperinflammation.^110^ Remarkably, P4 inhibits autophagy and the mTOR signaling pathway^111^ to attenuate this inflammatory signaling during SARS‐CoV‐2 infection.

On the other hand, Covid‐19 is associated with neuropsychiatric manifestations including confusion, headache, seizure, stroke, and dysautonomia due to alteration of brain neurotransmitters, mainly GABA inhibition and development of sympathetic storm. It has been revealed that P4 active metabolite allopregnanolone is regarded as a neurosteroid that acts as a positive modulator of GABA_A_.^40^ Cutler et al.^112^ in an experimental study, found that P4 therapy restored behavioral effects following traumatic brain injury by activation of GABA_A_ neurotransmission. Therefore, P4 therapy may reduce neuropsychiatric manifestations and dysautonomia in COVID‐19 patients through modulation of the central GABA pathway.

Moreover, P4 is regarded as an allosteric modulator of the nAchR.^16^ An experimental study found that P4 activates central nAchR leading to neuroprotective effects.^113^ In COVID‐19, SARS‐CoV‐2 binds nAchR leading to dysregulation of the anti‐inflammatory cholinergic system with further augmentation of the release of pro‐inflammatory cytokines.^114^ Changeux et al.^115^ found that nicotine can compete with SARS‐CoV‐2 in binding nAchR, so nicotine is regarded as a therapeutic and preventive agent against SARS‐CoV‐2. P4 through stimulation of nAchR^40^ may prevent the interaction between SARS‐CoV‐2 and nAchR with attenuation of SARS‐CoV‐2‐induced inflammatory reactions. Further, P4 restores autonomic nervous system balance through the activation of nAchR, preventing the development of a sympathetic storm in COVID‐19.^116^

**Figure 4:**
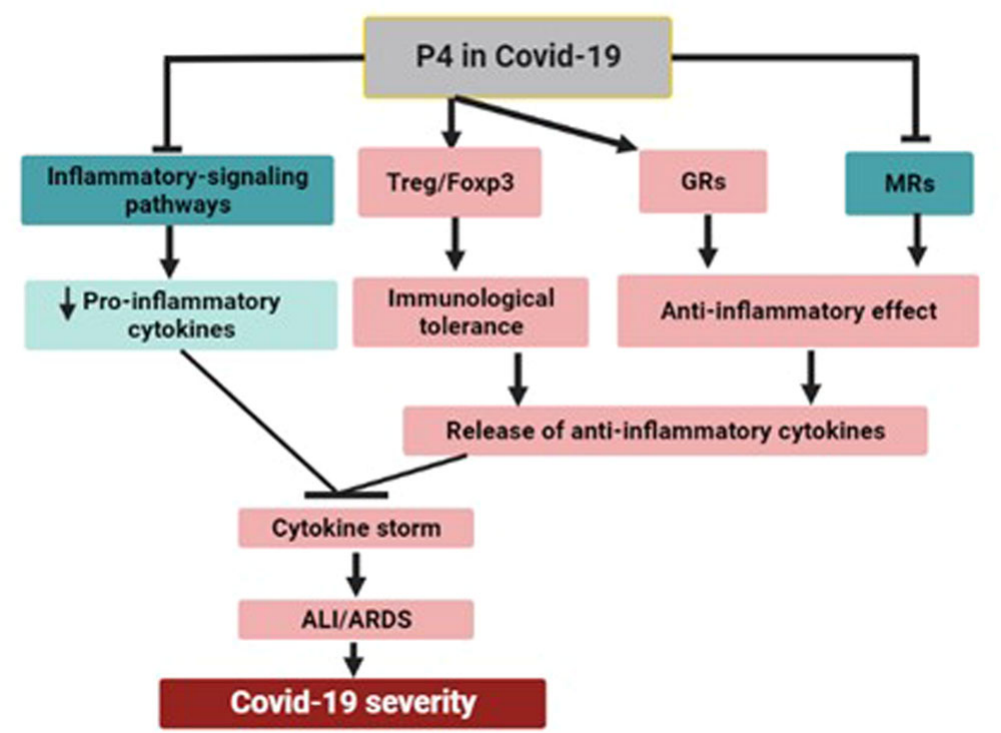
Progesterone role in Covid‐19: Progesterone (P4) inhibits inflammatory signaling pathways and mineralocorticoid receptors (MRs), activates regulatory T cells (Treg)/forkhead box P3 (FOXP3) and glucocorticoid receptors (GRs), leading to activation release of anti‐inflammatory cytokines and inhibition release of pro‐inflammatory cytokines with inhibition development of cytokine storm. These changes inhibit the development of acute lung injury (ALI) and acute respiratory distress syndrome (ARDS), reducing Covid‐19 severity.

Therefore, P4, through modulation of inflammatory signaling pathways, pro‐inflammatory cytokines, anti‐inflammatory cytokines, immunological tolerance, and GABA_A_ neurotransmission, may improve central and peripheral complications in COVID‐19 (Figure 4).

The present narrative review study had several limitations, including the paucity of clinical studies regarding the use of P4 in treating COVID‐19. Most of the mechanisms related to the potential effects of P4 on Covid‐19 are speculative depending on previous experimental and clinical conditions studies. However, this study opens a new way regarding the beneficial effects of P4 in the management of COVID‐19 that need to be confirmed by clinical trials and prospective studies.

## 3 CONCLUSION

P4 has anti‐inflammatory and immunosuppressive effects; thereby, it is effective in different inflammatory disorders. The regulatory effects of P4 on various viral infections are controversial as it may reduce or ameliorate these viral infections. However, the possible role of P4 in COVID‐19 could be beneficial through the modulation of inflammatory signaling pathways, induction of the release of anti‐inflammatory cytokines, and inhibition release of pro‐inflammatory cytokines. However, P4 is capable of shifting the immune response from an anti‐viral Th1 response to a Th2 response, which is likely to favor the virus. This effect may reduce the risk of SARS‐CoV‐2‐induced cytokine storm, but may enhance the proliferation of SARS‐CoV‐2. Therefore, the role of P4 could be beneficial or detrimental to the course of SARS‐CoV‐2 infection and COVID‐19 severity. This review cannot conclude the final role of P4 in COVID‐19. Herein, clinical trials and prospective studies are recommended to substantiate the potential role of P4 in the management of COVID‐19 regarding sex and the reproductive period.

## AUTHOR CONTRIBUTIONS

**Hayder M. Al‐Kuraishy**: conceptualization; resources. **Thabat J. Al‐Maiahy**: conceptualization; resources. **Ali I. Al‐Gareeb**: conceptualization; resources. **Athanasios Alexiou**: writing—original draft. **Marios Papadakis**: funding acquisition; writing—review & editing. **Omnya Elhussieny**: writing—review & editing. **Hebatallah M. Saad**: supervision; writing—review & editing. **Gaber El‐Saber Batiha**: validation; visualization; writing—original draft.

## CONFLICT OF INTEREST STATEMENT

The authors declare no conflicts of interest.

## Data Availability

Not applicable.
